# Interaction of Brain-Derived Neurotrophic Factor with the Effects of Chronic Methamphetamine on Prepulse Inhibition in Mice Is Independent of Dopamine D3 Receptors

**DOI:** 10.3390/biomedicines11082290

**Published:** 2023-08-17

**Authors:** Samuel Hogarth, Emily J. Jaehne, Xiangjun Xu, Quenten Schwarz, Maarten van den Buuse

**Affiliations:** 1 School of Psychology and Public Health, La Trobe University, Melbourne, VIC 3086, Australiae.jaehne@latrobe.edu.au (E.J.J.); 2 Centre for Cancer Biology, University of South Australia, Adelaide, SA 5000, Australiaquenten.schwarz@unisa.edu.au (Q.S.)

**Keywords:** prepulse inhibition, methamphetamine, brain-derived neurotrophic factor, sex differences, dopamine D3 receptors, striatum, frontal cortex

## Abstract

The aim of the present study was to gain a better understanding of the role of brain-derived neurotrophic factor (BDNF) and dopamine D3 receptors in the effects of chronic methamphetamine (METH) on prepulse inhibition (PPI), an endophenotype of psychosis. We compared the effect of a three-week adolescent METH treatment protocol on the regulation of PPI in wildtype mice, BDNF heterozygous mice (HET), D3 receptor knockout mice (D3KO), and double-mutant mice (DM) with both BDNF heterozygosity and D3 receptor knockout. Chronic METH induced disruption of PPI regulation in male mice with BDNF haploinsufficiency (HET and DM), independent of D3 receptor knockout. Specifically, these mice showed reduced baseline PPI, as well as attenuated disruption of PPI induced by acute treatment with the dopamine receptor agonist, apomorphine (APO), or the glutamate NMDA receptor antagonist, MK-801. In contrast, there were no effects of BDNF heterozygosity or D3 knockout on PPI regulation in female mice. Chronic METH pretreatment induced the expected locomotor hyperactivity sensitisation, where female HET and DM mice also showed endogenous sensitisation. Differential sex-specific effects of genotype and METH pretreatment were observed on dopamine receptor and dopamine transporter gene expression in the striatum and frontal cortex. Taken together, these results show a significant involvement of BDNF in the long-term effects of METH on PPI, particularly in male mice, but these effects appear independent of D3 receptors. The role of this receptor in psychosis endophenotypes therefore remains unclear.

## 1. Introduction

Methamphetamine (METH; N-methyl-alpha-methylphenethylamine) is a widely abused psychostimulant drug that can induce significant and long-lasting neuroplastic and neurotoxic effects on the mammalian brain [1,2] and may cause psychosis similar to paranoid schizophrenia [3]. Chronic METH use also causes behavioural sensitisation [1], the progressive enhancement of its effect following repeated exposure. This sensitisation can persist for weeks following METH withdrawal [4]. Dopaminergic sensitisation has been proposed as a key mechanism mediating subcortical hyperdopaminergia in both schizophrenia and METH psychosis [5,6,7,8]. Investigation of the long-term effect of repeated METH treatment may therefore inform on the neurochemical mechanisms involved in clinical psychosis and schizophrenia.

Brain-derived neurotrophic factor (BDNF) is an abundant growth factor involved in neuroplasticity and development of the nervous system [9,10]. Deficient BDNF signalling is implicated in the development of several pathophysiologies in neuronal populations [11], particularly within mesocorticolimbic dopamine pathways associated with reward, pleasure, executive function [1,12], as well as addiction [1,13,14,15] and neuropsychiatric conditions [16,17,18,19]. BDNF heterozygous mutant mice and rats (HET) carry a null mutation in one BDNF gene allele, conferring a 50% reduction in endogenous protein production [20,21]. HET have been used extensively in determining the extent of neuronal dysfunction instigated by reduced trophic signalling. Previous studies in these haploinsufficient rodents have begun to reveal how BDNF signalling is involved in the regulation of reward and relapse behaviours, such as sensitisation to cocaine [1,17,22]. BDNF HET mice also display increased sensitivity to the hyperlocomotion induced by drugs that increase extracellular dopamine release, such as amphetamine derivatives [23,24]. However, despite growing evidence for a role of BDNF in the development of addiction and psychosis, little progress has been made in determining the mechanisms involved in the contribution of BDNF to these diseases and how attenuated BDNF signalling impacts dopamine-driven behaviours.

BDNF has particularly been linked to the expression of the D3 receptor [25]. Although D3 receptor expression is lower than D2 receptor expression, D3 receptors are present on all mesencephalic dopaminergic neurons, presumably acting as a negative feedback autoreceptor to dopaminergic signalling. The activation of postsynaptic D3 receptors has furthermore been hypothesized to put a “brake” on hyperdopaminergic activity in the reward network, particularly within the D3 receptor-rich nucleus accumbens [26]. D3 knockout mice showed a heightened susceptibility to the effects of amphetamines, double the extracellular dopamine concentration of wildtype animals, and reduced dopamine reuptake from loss of dopamine transporter (DAT) regulation [27,28,29]. These mice are more susceptible and had increased responses to cues associated with opiates, cocaine, and amphetamines [28]. D3 receptor mRNA has been detected in areas relevant to psychosis development including the ventral tegmental area (VTA), substantia nigra (SN), nucleus accumbens (NAc), and hippocampus in mice [30]. Consequently, D3 receptor dysfunction has been implicated in pathologies of drug addiction, depression, Parkinson’s disease, and schizophrenia [28]. Lower D3 receptor mRNA and protein expression has been reported in schizophrenia patients during episodes of acute psychosis and in chronic disease pathology [31,32,33], and some D3 receptor polymorphisms have been associated with schizophrenia [34,35].

Animal model studies have aimed to elucidate how BDNF promotes neuronal adaptations in target postsynaptic neurons via modulation of D3 receptors [29,36]. D3 receptor expression is reduced in BDNF HET mice [24], and BDNF from corticostriatal neurons induces behavioural sensitisation, most likely by triggering overexpression of the D3 receptor in the striatum [36]. However, the involvement of D3 receptors in the role of BDNF in psychosis progression and the mechanisms through which perturbed signalling occurs, remain largely unknown.

The aim of the present study was to gain a better understanding of the role of BDNF and D3 receptors in the effects of chronic METH on endophenotypes of psychosis. We used a chronic, three-week adolescent METH treatment protocol in BDNF HET mice [17,37] and investigated the role of dopamine D3 receptors in its effects. To this end, we developed a novel double-mutant mouse model with four possible genotypes: wildtype, D3 knockout (D3KO), BDNF HET, and double-mutant mice (DM) with both D3 knockout and BDNF HET. This allowed us to study the effect of BDNF deficiency on its own or in the presence of additional deletion of D3 receptors, as well as the effect of D3 receptor deletion on its own or in the presence of BDNF deficiency. Because several previous studies have shown profound sex differences in psychosis-like behaviour [38,39], we used both male and female mice.

In this study, we focused on prepulse inhibition (PPI), a model of sensorimotor gating associated with psychosis [39,40,41]. We assessed both baseline PPI and its disruption by dopaminergic stimulation or glutamate NMDA receptor blockade, mimicking hyperdopaminergia and NMDA receptor hypofunction, respectively [39,42,43,44]. A study in mice found no significant difference between D3 knockout mice and wildtype mice for baseline PPI or PPI disruption caused by acute treatment with amphetamine [45]. However, D3 knockout mice were more susceptible to the PPI-disrupting effects of acute cocaine administration, displaying greater gating deficits [46]. We also verified METH-induced sensitisation by assessment of locomotor hyperactivity induced by an acute challenge with METH several weeks after the chronic pretreatment protocol [17,39]. To elucidate dopaminergic plasticity markers potentially involved in the behavioural effects, finally we determined the expression of dopamine D1, D2, and D3 receptors, as well as that of the dopamine transporter (DAT), in the striatum and frontal cortex, brain regions implicated in sensitisation and psychosis.

## 2. Materials and Methods

### 2.1. Mice

Male and female mice were bred and housed at the La Trobe Animal Research and Teaching Facility (LARTF; La Trobe University, Melbourne, Australia). These included wildtype controls (WT), BDNF HET, D3KO, and double-mutant mice (DM) with both D3KO and BDNF haploinsufficiency. BDNF HET mice were originally established through a targeted deletion of the BDNF gene [47]. Because of the limited viability of homozygote BDNF knockout mice [48], after backcrossing onto the C57Bl/6 genetic background, this line was maintained via wildtype × HET crosses. We previously confirmed that BDNF HET mice have an approximate 50% reduction in BDNF protein [21]. D3 knockout mice were a generous gift of Dr John Drago, University of Melbourne, Australia, and were originally established by targeted deletion of the mouse D3 receptor gene [49,50] in the 129/sv strain prior to backcrossing onto a C57BL/6 genetic background. BDNF HET/D3KO double-mutant mice were bred via a two-step breeding strategy (see also Supplementary Figure S1):

Step 1: Crossing of BDNF HET with D3 knockout mice, generating 50% BDNF HET/D3 HET and 50% BDNF wildtype/D3 HET mice with identical genetic background. These animals were used as founding breeders of the double-mutant line.
Step 2: Mating BDNF HET/D3 receptor HET mice with BDNF wildtype/D3 receptor HET, generating eight possible genetic combinations of which four were used in the present study (Supplementary Figure S1): WT, BDNF HET, D3KO and DM. The numbers of mice per group are shown in Table 1. For both breeding strategy steps, the genotype of male and female breeders was randomised to account for possible differences in maternal behaviour.

Mice were housed in individually ventilated cages (IVC; Tecniplast, Buguggiate, Italy) in same-sex groups of 2–5 and were provided with a stage and a cardboard tunnel as standard enrichment material. To account for possible litter effects, the number of mice per litter per adolescent treatment did not exceed two per experimental group. No attempts were made to determine the oestrus cycle stage of the female mice as this would have possibly added a level of stress to the protocol. Therefore, it was assumed that the oestrus cycle stage was random during treatment and behavioural testing. Mice had ad libitum access to standard rodent chow and drinking water and were maintained on a 12-h light/dark cycle, 8 am on, 8 pm off. Cages were cleaned every fortnight and all experiments were conducted during the light period. BDNF HET, D3KO, double-mutant, and WT mice were genotyped by Transnetyx (Cordova, TN, USA).

All experiments were approved by the La Trobe University Animal Ethics Committee and were conducted in accordance with the Australian Code of Practice for the Care and Use of Animals enacted by the National Health and Medical Research Council of Australia (NHMRC).

### 2.2. Chronic METH Treatment

During adolescence at 6 ± 0.5 weeks of age, mice were randomly assigned to receive METH ((±)-Methamphetamine-HCl, National Measurement Institute, Pymble, NSW, Australia), or a volume-matched saline vehicle solution (0.9% sodium chloride) to act as control. METH and saline solutions were administered via intraperitoneal (IP) injection at a volume of 5 mL/kg. Mice were administered 1 mg/kg METH Mon–Fri in the first week, 2× daily 2 mg/kg METH in the second, and 2× daily 4 mg/kg in the third week (Supplementary Figure S2) [17,37,51,52,53]. Saline-matched controls received the same frequency of injections. Animal weights were measured twice weekly to ensure accurate dosage across the five-day injection week. The body weight data reported here (Supplementary Figure S3) are for the start of the treatment period, the end of the treatment period, the end of the two-week no-treatment phase, and the end of behavioural testing. The escalating dose design in these studies covers a three-week window of adolescence in mice that overlaps with adolescence/young adulthood in humans, a period associated with enhanced vulnerability to the effects of psychotropic drugs and psychosis development [54].

None of the groups showed any detrimental effects of the chronic IP treatment on health and development reflected by body weight loss (Supplementary Figure S3). All mice increased their body weight over the course of the treatment and behavioural testing period (main effect of time, F(2.002, 354.3) = 1308.5, *p* < 0.001, ηp^2^ = 0.881), and male mice were heavier and gained more weight than female mice (main effect of sex, F(1, 177) = 284.9, *p* < 0.001, ηp^2^ = 0.617; sex × time interaction, F(2.002, 354.3) = 21.94, *p* < 0.001, ηp^2^ = 0.110). BDNF HET and double-mutant mice had higher body weights and greater body weight gain than wildtype and D3 knockout mice (main effect of combined BDNF genotype, F(1, 177) = 77.27, *p* < 0.001, ηp^2^ = 0.304; BDNF genotype × time interaction, F(2.002, 354.3) = 75.30, *p* < 0.001, ηp^2^ = 0.298). There were minor effects of METH pretreatment on body weight gain dependent on the BDNF genotype (Supplementary Figure S3).

### 2.3. Behavioural Analysis

Following the three-week chronic METH treatment, mice were left untreated for two weeks to enable METH washout and for long-lasting sensitisation to develop (Supplementary Figure S2). The animals were subsequently tested in four PPI sessions and three METH-induced locomotor hyperactivity sessions.

#### 2.3.1. Prepulse Inhibition

Mice were placed in Perspex cylinders within San Diego Instruments SR-Lab automated startle boxes (San Diego, CA, USA). A protocol of 104 trials was used over 40–45 min per session. Every session began with a 3-min 65 decibel (dB) background noise phase, followed by eight 115 dB pulse-alone startle stimuli. This block of pulse-alone stimuli was repeated at the end of the PPI session. Within each session, 16 additional pulse-alone trials were pseudorandomly inserted throughout the experiment and startle responses for each mouse were calculated over four blocks of these pulse-alone stimuli to establish habituation (not shown here). The analysis of PPI was conducted on the average of the 32 pulse-alone trials.

A prepulse trial was defined as a prepulse stimulus (PP) of 2, 4, 8, or 16 dB above background that preceded the 115 dB pulse at an interstimulus interval (ISI) of 30 ms or 100 ms prior to the startle noise. In total, eight trials were pseudorandomly conducted for each PP intensity at both ISIs. A further eight no-stimulus trials were pseudorandomised within each session to determine any nonspecific motor effects or background vibration. PPI was low and more variable at the 30 ms ISI than the 100 ms ISI and generally did not differ across sex or pretreatment groups; therefore, data presentation focuses on the more commonly used 100 ms ISI.

Startle responses were measured through vibrations detected by the PPI chamber and relayed to SR_Lab software (version 2006–2010, San Diego Instruments, San Diego, CA, USA) for analysis. PPI is a percentage value calculated by the difference between responses to the eight pulse-alone startle stimuli compared to responses to the pulses preceded by one of the four prepulses.

Four PPI sessions were conducted in each mouse with a 2–3 day washout between experiments: (1) A pretest with no injection to acclimatize the mice to the PPI chamber; (2) a saline IP injection directly prior to the PPI session; (3) 3 mg/kg APO (R-(−)-Apomorphine, Sigma Aldrich, Castle Hill, NSW, Australia) injected IP directly prior to the PPI session; (4) 0.2 mg/kg MK-801 ((+)-MK-801 hydrogen maleate, Sigma Aldrich, Castle Hill, NSW, Australia) injected IP 15 min prior to the PPI session. These drugs were selected due to their capacity to disrupt PPI via neurochemical pathways relevant to schizophrenia, particularly dopamine (APO) and NMDA receptor hypofunction (MK-801) [39]. The order of saline, APO, and MK-801 injection sessions was pseudorandomised.

#### 2.3.2. Locomotor Hyperactivity

Five to seven days after the last PPI session, mice were tested for baseline and METH-induced locomotor hyperactivity using automated locomotor photocells (Seamless Open Field Arena, Med Associates, Inc., Fairfax, VT, USA). For each 180 min session, mice were placed individually into these photocell chambers and allowed to habituate for 60 min. Between the 55 and 60 min time point, each mouse received an acute IP injection before being returned to the photocell where activity was further recorded over the following 120 min. Mice received saline vehicle in the first session, 1 mg/kg METH in the second session, and 3 mg/kg METH in the final session. Three to four days was left between sessions to allow drug washout. Photocell chambers recorded locomotion via the interruption of photoelectric beams positioned along the walls of the chamber. Mouse activity was recorded and analysed by the Activity Monitor software (version 7.0.5.10, Med Associates Inc., Fairfax, VT, USA), and outputs for the statistical analysis included cumulative horizontal distance moved every 5 min.

### 2.4. Quantitative Reverse Transcriptase-PCR

At least one week after the last behavioural test, the mice were euthanised by CO_2_ inhalation and decapitation, and the frontal cortex and striatum were rapidly dissected on a cold plate. Samples were frozen on dry ice and stored at −80 °C until the PCR assay of dopamine D1, D2, and D3 receptors and DAT gene expression.

Total RNA was isolated from dissected tissue using Trizol (Ambion, Austin, TX, USA) and single-stranded complementary DNA was synthesized using the QuantiTect Reverse transcription kit (Qiagen, Frankfurt, Germany). Quantitative PCR was performed with an SYBR Green reagent (Qiagen, Frakfurt, Germany) using the Rotor-Gene 6000 real-time PCR system (Corbett Life Science, Frankfurt, Germany). The primers used were:

*beta actin:* F: 5′-GATCATTGCTCCTCCTGAGC-3′, R: 5′-AGTCCGCCTAGAAGCACTTG-3′
*D1 receptor:* F: 5′-CCAGATCGGGCATTTGGAGA-3′, R: 5′-GGGCCTCTTCCTGGTCAATC-3′
*D2 receptor:* F: 5′-GTCTCGTTCTACGTGCCCTT-3′, R: 5′-GGTGGGTACAGTTGCCCTTG-3′
*D3 receptor:* F: 5′-ACTTGGAGGTGACAGGTGGA-3′, R: 5′-GGCATGACCACTGCTGTGTA-3′
*DAT:* F: 5′-CCTGGTTCTACGGTGTCCAG-3′, R: 5′-GCTGACCACGACCACATACA-3′.

Relative mRNA levels were quantified using the delta–delta CT method (normalised to *beta-actin*). Each PCR was performed in technical triplicate. Error bars represent the standard error of the mean (SEM) between biological replicates.

### 2.5. Data Analysis

PPI increased with prepulse intensity dB levels. We did not observe any statistical interactions between prepulse intensity level and either genotype, sex, or acute drug treatment. Similarly, startle habituation did not interact with these factors. Therefore, for clarity, figures only show average PPI and average startle values. PPI data separated by prepulse intensity are available in the supplementary results (Supplementary Table S1).

All statistical analysis was conducted using IBM SPSS (Statistics for Windows, Version 26.0. IBM Corp., Armonk, NY, USA). Outliers were detected using Z scores. Analysis of variance (ANOVA) statistical models were used to examine the effects of between-subject variables such as genotype, sex, and pretreatment. Because of technical limitations, PCR gene expression assay was done separately in samples from male and female mice. Therefore, the statistical analysis could not include a direct male/female comparison but instead focused on genotype and METH pretreatment effects in males and females separately. When significant differences were observed between the genotypes (four groups: wildtype, D3KO, BDNF HET, DM), the analysis of genotype effects was split into D3 receptor subgroups (WT and BDNF HET vs. D3KO and DM) or BDNF genotype subgroups (WT and D3KO vs. BDNF HET and DM) to determine potential genotype interactions and relative contributions to behaviour.

Where applicable, a repeated-measures ANOVA was used to analyse within-subject factors in mice’ behavioural data. These included prepulse intensity and drug effects for PPI, time bin analyses and drug effects for locomotor hyperactivity, and change in body weight over the course of the experiment. In cases where data were not normally distributed and sphericity could not be assumed, as demonstrated by a significant Mauchly’s test, the Greenhouse-Geisser correction was used.

ANOVA results were considered statistically significant when *p* < 0.05. Effect size was expressed as partial eta squared (ηp^2^). Data in all graphs are represented as the mean ± standard error of the mean (SEM).

## 3. Results

### 3.1. Baseline PPI and Startle Are Affected by BDNF Genotype but Not by D3 Receptor Deletion

**Figure 1:**
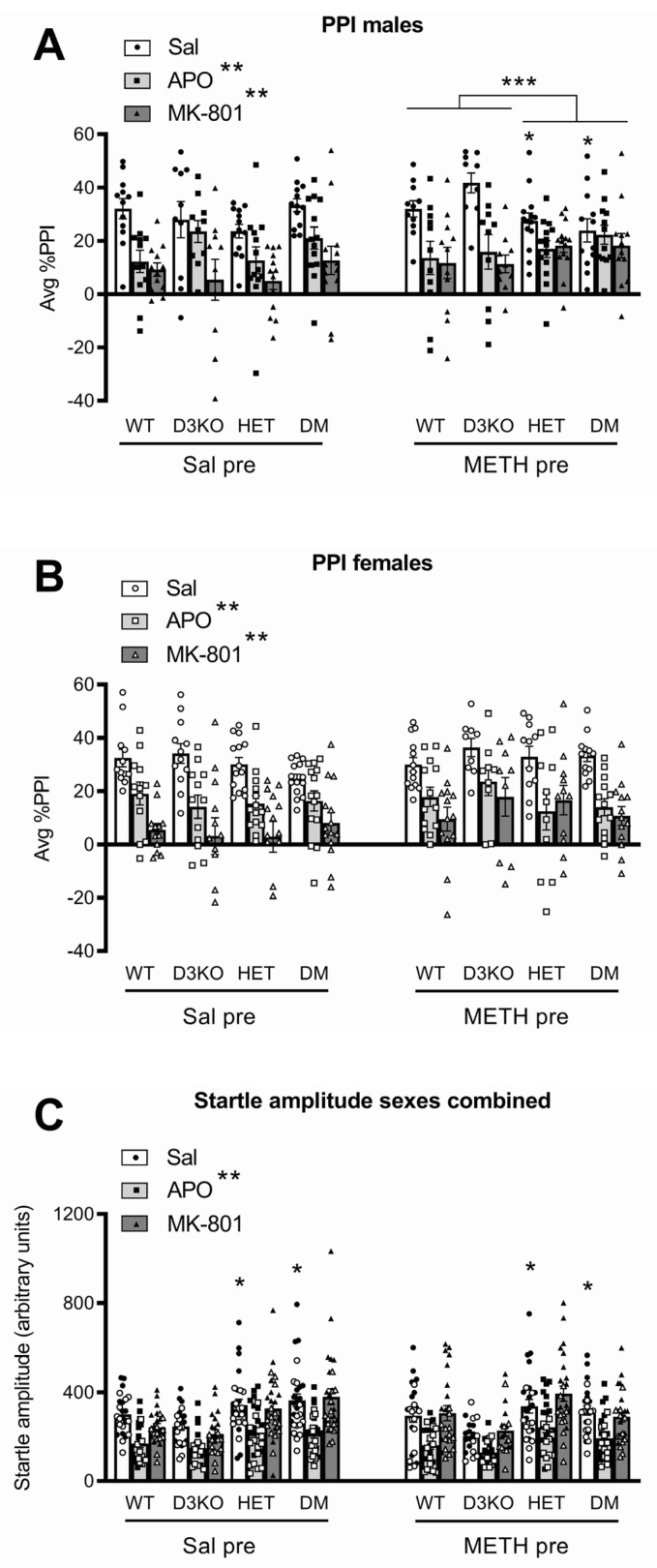
Average prepulse inhibition (PPI) in male (**A**) and female (**B**) wildtype (WT), D3 receptor knockout (D3KO), BDNF heterozygous mice (HET), and double-mutant mice with both BDNF heterozygosity and D3 knockout (DM). The mice were pretreated with a three-week injection regimen of methamphetamine (METH) or saline. Two weeks later, PPI was measured at baseline following saline injection (Sal) while PPI disruption was induced by an acute challenge with apomorphine (APO) or MK-801 (** *p* < 0.05). Following METH pretreatment, baseline PPI (* *p* < 0.05) and the effects of APO and MK-801 (*** *p* < 0.05) were significantly reduced in male HET and DM mice compared to WT and D3KO mice. APO caused a reduction of startle amplitudes but there were no genotype differences or effects of METH on startle amplitudes (**C**).

Baseline PPI was measured following acute saline injection. Average PPI was significantly affected by genotype (F(3, 173) = 4.30; *p* = 0.006, ηp^2^ = 0.069), and there was a significant three-way interaction between sex, METH pretreatment, and genotype (F(3, 173) = 3.09, *p* = 0.028, ηp^2^ = 0.051). To explore this interaction, the data were split by pretreatment and sex and analysed for an effect of the BDNF genotype or D3 receptor genotype (Figure 1A,B). Baseline PPI in male mice pretreated with saline did not differ significantly between either the BDNF of D3 receptor genotypes (Figure 1A). In contrast, after pretreatment with METH, PPI was lower in male mice with either of the BDNF genotypes (F(1, 43) = 9.69, *p* = 0.003, ηp^2^ = 0.184), but there was no D3 genotype effect or interaction between the two genotypes. There were no genotype differences in female mice (Figure 1B).

Baseline startle amplitude was significantly lower in female mice compared to male mice (main effect of sex, F(1, 173) = 28.62, *p* < 0.001, ηp^2^ = 0.142, not shown). A significant main effect of genotype (F(3, 173) = 10.09; *p* < 0.001, ηp^2^ = 0.149) prompted further analysis, in which post hoc comparison identified that, independent of METH pretreatment, BDNF HET and DM mice had significantly higher startle compared to D3 knockout mice but not wildtype mice (*p* < 0.05; Figure 1C).

### 3.2. BDNF Haploinsufficiency, but Not D3 Receptor Knockout, Reduces APO-Induced PPI Disruption in METH-Sensitised Male Mice

Compared to saline IP injection, acute APO administration significantly reduced PPI (F(1, 173) = 115.4, *p* < 0.001, ηp^2^ = 0.400; Figure 1). A significant four-way interaction (APO × sex × genotype × pretreatment, F(3, 173) = 3.19, *p* = 0.025, ηp^2^ = 0.052) prompted further investigation of the data split by sex and pretreatment and analysed for an effect of the BDNF genotype or D3 receptor genotype (Figure 1A,B). In male mice, APO-induced PPI disruption was affected by the BDNF genotype depending on METH pretreatment. Compared to WT and D3KO, METH-pretreated male BDNF HET and DM mice had an attenuated APO response (interaction of BDNF HET × APO, F(1, 43) = 9.41; *p* = 0.004, ηp^2^ = 0.179) but there was no interaction with the D3 receptor genotype (Figure 1A). There were no genotype differences in the PPI response to acute APO treatment in male mice pretreated with saline (Figure 1A), nor in either saline- or METH-pretreated female mice (Figure 1B). Compared to baseline startle following saline injection, startle reactivity following the APO challenge was significantly attenuated (main effect APO, F(1, 173) = 204.34; *p* < 0.001, ηp^2^ = 0.542), independent of genotype, pretreatment, or sex (Figure 1C).

### 3.3. BDNF Haploinsufficiency, but Not D3 Receptor Knockout, Reduces MK-801-Induced PPI Disruption in METH-Sensitised Male Mice

Compared to acute saline IP injection, acute MK-801 administration significantly reduced PPI (F(1, 173) = 237.72, *p* < 0.001, ηp^2^ = 0.579; Figure 1). A significant genotype interaction was detected (MK-801 × genotype, F(3, 173) = 3.35, *p* = 0.02, ηp^2^ = 0.055) although the four-way interaction of MK-801 × sex × genotype × pretreatment did not reach statistical significance. However, ANOVA with the genotype split by BDNF heterozygosity or D3 knockout showed that, similar to the APO PPI data, male BDNF HET and DM mice pretreated with METH displayed a significantly attenuated PPI disruption compared to male WT and D3 knockout mice (F(1, 43) = 12.02; *p* = 0.001, ηp^2^ = 0.218; Figure 1A). The genotype of saline-pretreated male mice did not affect MK-801-induced disruption of PPI (Figure 1A), and in female mice there were no significant differences in the effect of MK-801 across genotype or pretreatment regimens (Figure 1B).

Analysis of average startle amplitude between saline and MK-801 PPI sessions showed a significant sex effect (interaction of MK-801 × sex, F(1, 173) = 7.02, *p* = 0.009, ηp^2^ = 0.039; for simplicity shown as sexes combined in Figure 1C; see Supplementary Figure S4 for male and female data separated). Sex-specific analysis of startle amplitudes showed no effect of MK-801 and no impact of genotype in male mice (Supplementary Figure S4). In female mice, startle amplitudes following the MK-801 treatment were slightly but significantly lower than baseline values (MK-801 main effect, F(1, 88) = 8.70, *p* = 0.004, ηp^2^ = 0.090), an effect which depended on the genotype (MK-801 × genotype, F(3, 88) = 3.11, *p* = 0.030, ηp^2^ = 0.096). Further analysis grouping mice by BDNF genotype showed a significant interaction (F(1, 92) = 5.18, *p* = 0.025, ηp^2^ = 0.056), and post hoc analysis showed MK-801-induced decreases in startle amplitudes in WT and D3 knockout mice but not in BDNF HET mice and DM mice (Supplementary Figure S4).

### 3.4. Female BDNF HET Mice Show Endogenous Sensitisation, and Chronic METH Induces Further Long-Term Locomotor Sensitisation Independent of BDNF or D3 Receptor Genotype

**Figure 2:**
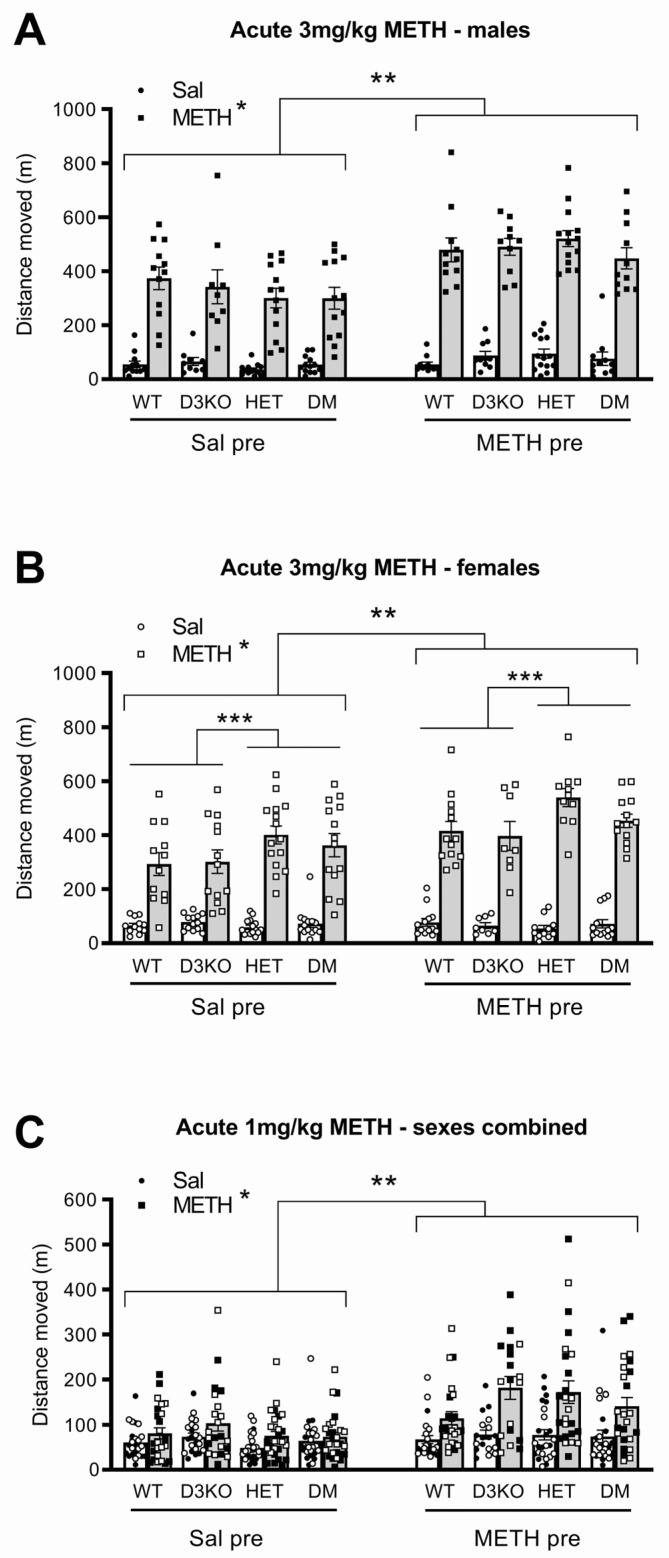
Locomotor hyperactivity induced by an acute METH challenge in male and female wildtype (WT), D3 receptor knockout (D3KO), BDNF heterozygous mice (HET), and double-mutant mice with both BDNF heterozygosity and D3 knockout (DM). The mice were pretreated with a three-week injection regimen of methamphetamine (METH pre) or saline (Sal pre). Locomotor activity was measured at baseline following saline injection (Sal) while hyperactivity (* *p* < 0.05) was induced by acute challenge with 3 mg/kg of METH (**A**,**B**) or 1 mg/kg of METH (**C**). Following METH pretreatment, the effect of an acute METH challenge was significantly enhanced in both male and female mice (** *p* < 0.05). Female HET and DM mice also showed endogenous sensitisation compared to WT and D3KO mice (*** *p* < 0.05). Acute 1 mg/kg METH produced modest locomotor hyperactivity (**C**), and this was enhanced (** *p* < 0.05) in mice that had received METH pretreatment, irrespective of sex or genotype in mice.

Compared to the baseline activity displayed after saline injection, a 3 mg/kg METH challenge induced significant locomotor hyperactivity (main effect of METH, F(1, 170) = 1136.71, *p* < 0.001, ηp^2^ = 0.870; Figure 2), and this hyperactivity was enhanced further by the METH pretreatment (interaction of METH × pretreatment, F(1, 170) = 35.52, *p* < 0.001, ηp^2^ = 0.173; Figure 2). A significant three-way interaction (METH × sex × genotype, F(3, 170) = 3.43, *p* = 0.018, ηp^2^ = 0.057) suggested sex-specific effects. There were no significant interactions of either the acute or pretreatment effects of METH with genotype in male mice.

In female mice, the effect of acute METH differed between genotypes (interaction of METH × genotype, F(3, 89) = 4.44, *p* = 0.006, ηp^2^ = 0.130), prompting further analysis by grouping the animals according to BDNF or D3 receptor genotype. Analysis of the effect of the D3 knockout genotype found no differences between mice with functional D3 receptors (WT and BDNF HET) and D3 knockouts (D3 knockout and DM mice). Conversely, analysis of the BDNF genotype found BDNF haploinsufficiency (BDNF HET and DM) significantly enhanced acute METH-induced locomotor hyperactivity compared to BDNF WT (WT and D3KO; interaction of METH × BDNF genotype, F(1, 89) = 10.46, *p* = 0.002, ηp^2^ = 0.105) independent of METH pretreatment (Figure 2B). This suggests female, but not male BDNF HET mice are endogenously sensitised to the locomotor-stimulating effects of METH.

An acute 1 mg/kg METH challenge produced modest locomotor hyperactivity (main effect of METH, F(1, 170) = 100.26, *p* < 0.001, ηp^2^ = 0.371; Figure 2C) and this was enhanced in mice that had received METH pretreatment (interaction of METH × pretreatment, F(1, 170) = 27.95, *p* < 0.001, ηp^2^ = 0.141), again suggesting sensitisation to the drug. No significant sex differences were observed, nor genotype differences, in the effect of the 1 mg/kg METH challenge (Figure 2C).

### 3.5. Sex-Specific Downregulation of Dopamine Receptor Gene Expression in BDNF HET Mice in the Striatum Independent of METH Pretreatment

**Figure 3:**
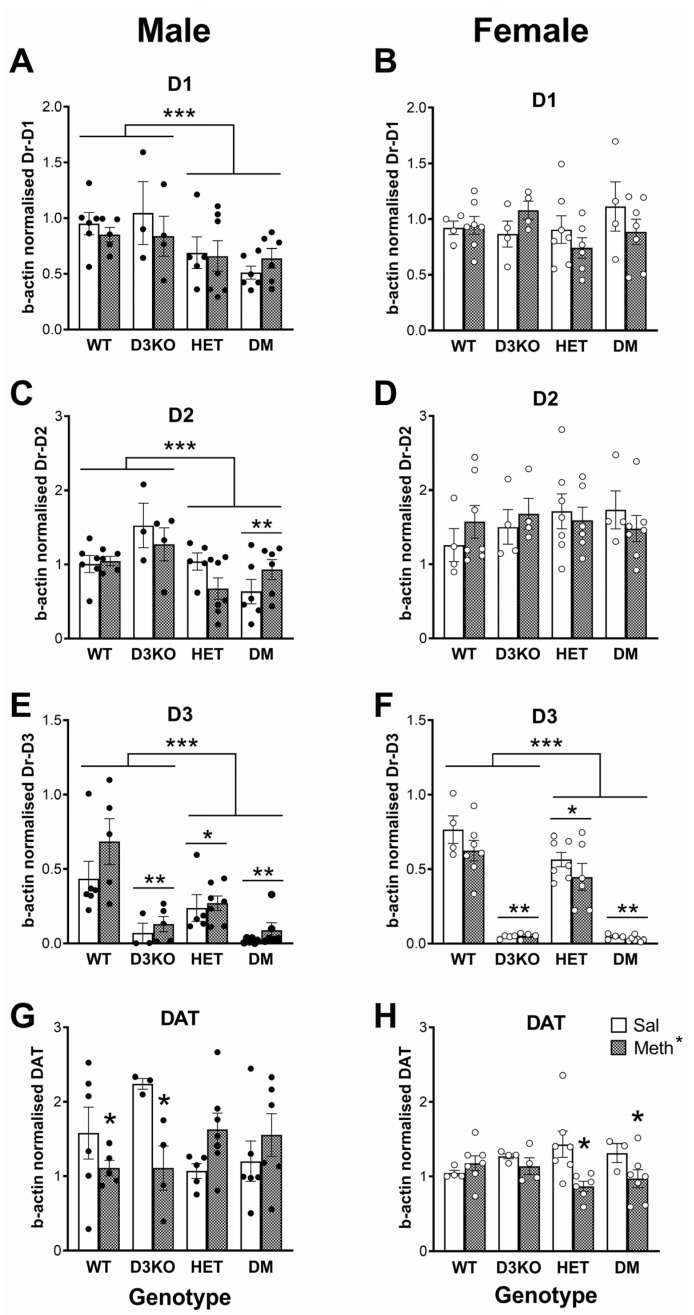
Expression of D1, D2, and D3 receptors (Dr) and dopamine transporters (DAT) in the striatum of male (left panels) and female (right panels) wildtype (WT), D3 receptor knockout (D3KO), BDNF heterozygous (HET), and double-mutant mice (DM) with both BDNF heterozygosity and D3 knockout. The mice were pretreated with a three-week injection regimen of methamphetamine (METH, filled bars) or saline (Sal, open bars). (**A**) D1 receptors in males; (**B**) D1 receptors in females; (**C**) D2 receptors in males; (**D**) D2 receptors in females; (**E**) D3 receptors in males; (**F**) D3 receptors in females; (**G**) DAT in males; (**H**) DAT in females. * *p* < 0.05 for the difference between METH-pretreated mice and saline-pretreated controls; ** *p* < 0.05 for the difference between D3KO genotype (D3KO and DM) vs. WT and HET mice; *** *p* < 0.05 for the difference between BDNF HET genotype (HET and DM) compared to WT and D3KO.

PCR analysis of dopamine D1 receptor gene expression in the striatum of male mice (Figure 3A) revealed significant differences between the groups (F(3, 34) = 3.83, *p* = 0.018, ηp^2^ = 0.252) with further comparisons of the combined genotype groups showing a significantly lower expression in BDNF HET and DM mice compared to wildtype and D3KO (F(1, 34) = 10.64, *p* = 0.003, ηp^2^ = 0.238). This difference was not seen in female mice (Figure 3B). Similarly, D2 receptor gene expression in males (Figure 3C) was different between the groups (F(3, 34) = 5.17, *p* = 0.005, ηp^2^ = 0.313), an effect which could be attributed to a lower expression in BDNF HET and DM mice compared to wildtype and D3KO mice (F(1, 34) = 12.61, *p* = 0.001, ηp^2^ = 0.271). There was also a pretreatment × BDNF genotype × D3 receptor genotype interaction (F(1, 34) = 4.67, *p* = 0.038, ηp^2^ = 0.121). Further comparison of the data from wildtype and BDNF HET mice showed no differences between the groups. In contrast, irrespective of METH pretreatment, DM mice had slightly lower D2 receptor gene expression than mice with D3 receptor knockout only (F(1, 15) = 10.02, *p* = 0.006, ηp^2^ = 0.400). There were no differences in D2 receptor expression between the groups in female mice (Figure 3D).

As expected, dopamine D3 receptor expression (Figure 3E) was significantly reduced in male D3KO and DM mice (main effect of group, F(3, 35) = 15.1, *p* < 0.001, ηp^2^ = 0.565; main effect of D3 receptor genotype, F(1, 35) = 29.9, *p* < 0.001, ηp^2^ = 0.461). In addition, D3 receptor gene expression significantly depended on the BDNF genotype (main effect of BDNF genotype, F(3, 35) = 8.48, *p* = 0.006, ηp^2^ = 0.195; BDNF genotype × D3 receptor genotype, F(1, 35) = 4.62, *p* = 0.039, ηp^2^ = 0.117). D3 receptor gene expression was significantly lower in male BDNF HET mice compared to wildtype mice (F(1, 19) = 8.76, *p* = 0.008, ηp^2^ = 0.316), but there were no differences between D3 knockouts and double-mutant mice, presumably because gene expression was already very low (Figure 3E). A similar pattern was observed in female mice (main effect of group, F(3, 35) = 58.2, *p* < 0.001, ηp^2^ = 0.833; main effect of D3 genotype, F(1, 35) = 167.5, *p* < 0.001, ηp^2^ = 0.827; main effect of BDNF genotype, F(1, 35) = 5.33, *p* = 0.027, ηp^2^ = 0.132; D3 genotype × BDNF genotype interaction, F(1, 35) = 4.35, *p* = 0.044, ηp^2^ = 0.111). D3 receptor expression was significantly lower in BDNF HET mice compared to wildtype mice (F(1, 20) = 6.31, *p* = 0.021, ηp^2^ = 0.240) but there were no genotype differences between the D3 knockout groups (Figure 3F).

### 3.6. Differential Effects of Chronic METH on DAT Expression in Striatum Dependent on Sex, D3 Receptor and BDNF Genotype

While there were no major effects of METH pretreatment on dopamine receptor expression, analysis of DAT expression in male mice (Figure 3G) showed group differences that were dependent on the pretreatment (interaction, F(3, 34) = 3.90, *p* = 0.017, ηp^2^ = 0.256). Separation of groups by BDNF genotype (METH × BDNF genotype interaction, F(1, 34) = 11.21, *p* = 0.002, ηp^2^ = 0.248) showed that the METH pretreatment significantly reduced DAT expression in wildtype and D3KO (F(1, 14) = 7.64, *p* = 0.015, ηp^2^ = 0.353), but BDNF HET and double-mutant mice tended to show an increase in DAT expression, although this did not reach statistical significance (*p* = 0.072). In contrast to male mice, in female mice METH pretreatment reduced DAT expression (main effect of pretreatment, F(1, 34) = 6.16, *p* = 0.018, ηp^2^ = 0.153) and this effect differed between the groups (pretreatment × group interaction, F(3, 34) = 3.09, *p* = 0.040, ηp^2^ = 0.214). This effect could be attributed to reduced DAT expression depending on the BDNF genotype (pretreatment × BDNF genotype, F(1, 34) = 6.16, *p* = 0.018, ηp^2^ = 0.153). A split of the data by BDNF genotype showed that METH pretreatment had no effect in wildtype mice and D3KO, but it significantly reduced expression in BDNF HET and DM mice (F(1, 19) = 9.42, *p* = 0.006, ηp^2^ = 0.331; Figure 3H)

### 3.7. Sex-Specific Downregulation of Dopamine Receptor Expression in BDNF HET Mice in the Frontal Cortex

**Figure 4:**
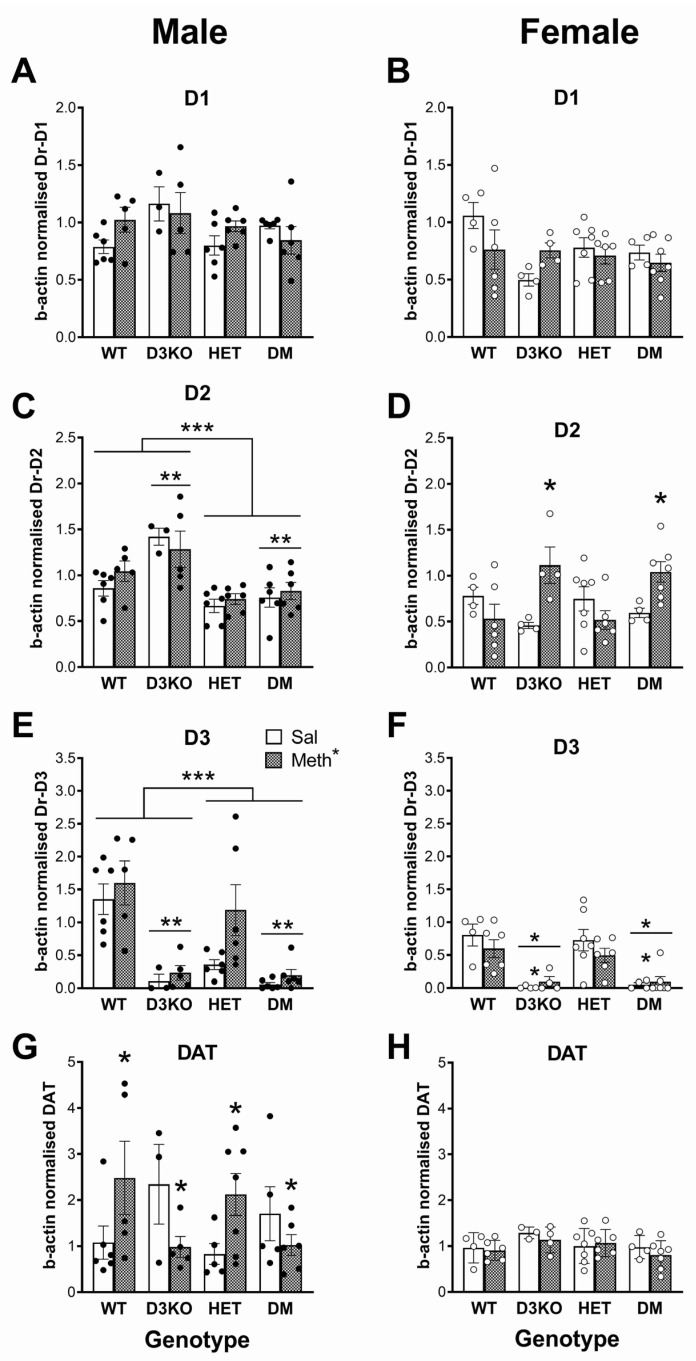
Expression of D1, D2, and D3 receptors (Dr) and dopamine transporters (DAT) in the frontal cortex of male (left panels) and female (right panels) wildtype (WT), D3 receptor knockout (D3KO), BDNF heterozygous mice (HET), and double-mutant mice (DM) with both BDNF heterozygosity and D3 knockout. The mice were pretreated with a three-week injection regimen of methamphetamine (METH, filled bars) or saline (Sal, open bars). (**A**) D1 receptors in males; (**B**) D1 receptors in females; (**C**) D2 receptors in males; (**D**) D2 receptors in females; (**E**) D3 receptors in males; (**F**) D3 receptors in females; (**G**) DAT in males; (**H**) DAT in females. * *p* < 0.05 for the difference between METH-pretreated mice and saline-pretreated controls; ** *p* < 0.05 for the difference between D3KO genotype (D3KO and DM) vs. WT and HET mice; *** *p* < 0.05 for the difference between BDNF HET genotype (HET and DM) compared to WT and D3KO.

PCR analysis of dopamine D1 receptor gene expression in the frontal cortex of males revealed no differences between the groups or any effects of the METH pretreatment (Figure 4A). A similar lack of genotype effects was found in female mice (Figure 4B). In contrast, analysis of D2 receptor expression in male mice showed significant differences between the groups (F(1, 35) = 11.8, *p* < 0.001, ηp^2^ = 0.503). Further analysis showed significantly reduced D2 receptor expression in BDNF HET mice and double-mutant mice compared to the combined wildtype and D3 knockout groups (main effect of BDNF genotype, F(1, 35) = 27.3, *p* < 0.001, ηp^2^ = 0.438) but a significant increase in D3 receptor knockout mice and double-mutant mice compared to wildtype and BDNF HET mice combined (main effect of D3 receptor genotype (F(1, 35) = 10.07, *p* = 0.003, ηp^2^ = 0.223). These effects were independent of METH pretreatment (Figure 4C). In contrast, in the frontal cortex of female mice there was a pretreatment × genotype interaction (F(3, 34) = 6.07, *p* = 0.002, ηp^2^ = 0.349). Split analysis of the data by genotype group showed that D2 receptor expression was upregulated by the METH pretreatment in female mice with the D3 receptor knockout genotype (F(1, 15) = 19.67, *p* < 0.001, ηp^2^ = 0.567) but there was no METH pretreatment effect in wildtype mice and BDNF HET mice (Figure 4D).

Similar to the striatum, D3 receptor gene expression in the frontal cortex was markedly and significantly reduced in male D3 knockout mice (main effect of group, F(3, 35) = 16.2, *p* < 0.002, ηp^2^ = 0.581; main effect of D3 genotype, F(1, 35) = 37.4, *p* < 0.001, ηp^2^ = 0.517). In addition, D3 receptor gene expression significantly depended on the BDNF genotype (main effect of BDNF genotype, F(1, 35) = 5.49, *p* = 0.025, ηp^2^ = 0.136; BDNF genotype × D3 receptor genotype, F(1, 35) = 4.28, *p* = 0.046, ηp^2^ = 0.109). D3 receptor gene expression was significantly lower in male BDNF HET mice compared to wildtype mice F(1, 19) = 6.30, *p* = 0.021, ηp^2^ = 0.249), but there were no differences between male D3 knockouts and double-mutant mice, presumably because gene expression was already very low (Figure 4E). While the genotype differences were independent of METH pretreatment, there was a small increase in D3 gene expression in METH-pretreated mice compared to saline-pretreated controls independent of the genotype (main effect, F(1, 35) = 4.41, *p* = 0.043, ηp^2^ = 0.112). In female mice, D3 receptor expression was different between the groups (main effect of group, F(3, 34) = 14.8, *p* < 0.001, ηp^2^ = 0.566) and D3 receptors were markedly downregulated in D3 knockout mice and double-mutant mice (F(1, 35) = 44.1, *p* < 0.001, ηp^2^ = 0.565), independent of the METH pretreatment (Figure 4F).

### 3.8. Differential Effects of Chronic METH on DAT Expression in Frontal Cortex Dependent on Sex and D3 Receptor Genotype

In male mice, DAT expression was altered by METH pretreatment depending on the group (Figure 4G, F(3, 34) = 3.93, *p* = 0.017, ηp^2^ = 0.257) and specifically the D3 receptor genotype (F(1, 34) = 11.7, *p* = 0.002, ηp^2^ = 0.257). In male mice with intact D3 receptor expression (wildtype and BDNF HET), METH pretreatment significantly increased DAT expression (F(1, 19) = 7.37, *p* = 0.014, ηp^2^ = 0.279). In contrast, in male mice lacking D3 receptors (D3 knockout and double-mutant mice), METH pretreatment reduced DAT gene expression (F(1, 15) = 4.98, *p* = 0.041, ηp^2^ = 0.249). There were no differences between the groups in the frontal cortex of female mice (Figure 4H).

## 4. Discussion

The aim of this study was to examine the potential interaction between BDNF and the D3 receptor in the long-term effects of METH on PPI in mice. The results show that METH sensitisation induces a disruption of PPI regulation in male mice with BDNF haploinsufficiency, independent of D3 receptor knockout. Specifically, these mice showed reduced baseline PPI, as well as attenuated disruption of PPI induced by acute treatment with APO or MK-801. In contrast, there were no effects of the BDNF HET or D3 knockout genotype on PPI regulation in female mice. Thus, intact BDNF signalling appears to normally protect particularly males from METH causing dysregulation of PPI. Chronic METH pretreatment induced the expected behavioural sensitisation and female BDNF HET and double-mutant mice also showed endogenous sensitisation. Sex-specific effects of genotype and METH pretreatment were observed on dopamine receptor and DAT gene expression in the striatum and frontal cortex. Taken together, these results show significant involvement of BDNF in the long-term effects of METH on PPI, particularly in male mice, independent of D3 receptors.

Behavioural sensitisation denotes the progressive and enduring enhancement of a behavioural response following repeated stimuli [1,6,8]. In humans, extended and repetitive use of psychostimulant drugs may lead to the development of sensitized behaviours characterised by a lasting hyper-responsiveness of midbrain dopaminergic neurons, auditory and visual hallucinations, paranoia, and psychomotor output [4,55]. Sensitisation of the dopaminergic neural circuitry is also hypothesized to play a role in schizophrenia pathophysiology and positive symptom progression [5,6,8]. A large body of evidence supports greater mesolimbic and nigrostriatal dopamine system reactivity in patients with schizophrenia and enhanced dopamine synthesis compared to unaffected controls [6,7,8]. These neuroadaptations form a state of endogenous sensitisation that is hypothesized to develop in both schizophrenia patients and following chronic METH use [1,5,8].

As BDNF is required for normal expression of the inhibitory D3 receptor [24,36,56], a reduction in BDNF expression could result in attenuated D3 receptor availability, promoting an enhanced rate of dopaminergic sensitisation. BDNF heterozygosity may therefore promote a state of endogenous sensitisation to dopamine-releasing agents as we have previously observed [17]. In the present study, we further investigated the interaction between D3 receptor expression and BDNF in behavioural sensitisation following METH administration, comparing wildtype, BDNF HET, D3KO, and double-mutant mice carrying both genetic alterations.

### 4.1. Chronic METH Affects Baseline PPI and Drug-Induced PPI Disruption in Male BDNF HET Mice Independent of D3 Receptors

METH-pretreated BDNF HET and DM male mice had lower average baseline PPI than the combined WT and D3 knockout group. These effects were not seen in METH-pretreated female or saline-pretreated mice. These data support previous findings of a reduced baseline PPI in BDNF HET mice [37], at least in male mice. The effect of APO and MK-801 to cause the expected PPI disruption, was absent in male BDNF HET and DM mice pretreated with METH, suggesting involvement of BDNF in METH-induced changes in dopaminergic pathways governing PPI in male mice. Specifically, METH-induced dopaminergic sensitisation in BDNF HET mice disrupted the efficacy of both D2/D1 receptor agonism and NMDA receptor antagonism in reducing PPI. It is possible that the reduction in baseline PPI in BDNF HET and DM mice may have affected the ability of APO and MK-801 to produce a further PPI reduction. However, while significantly lower than in WT and D3 knockout groups, baseline PPI in BDNF HET and DM mice was still around 15–20%, allowing further possible reduction by APO and MK-801, an effect which did not occur.

While the PPI-decreasing effect of APO and MK-801 is generally interpreted as modelling dopaminergic hyperactivity and glutamatergic hypoactivity, respectively, both purported to underlie positive symptoms of schizophrenia [39,41], the lack of effect of these drugs in our study may also represent a loss of the neurotransmitter regulation of PPI, as we discussed in a previous study using a developmental rat model of the illness [57]. There, following maternal immune activation, offspring showed reduced baseline PPI and reduced disruption of PPI after an acute APO challenge [57], similar to the present findings.

While baseline PPI and the effects of APO and MK-801 were reduced in male BDNF HET and DM mice, there were no such effects in female mice. This suggests a sex-specific vulnerability to the effect of deficient BDNF signalling, in this case unmasking an effect of chronic METH on PPI only in males. Previously, we observed a similar sex-specificity of the role of BDNF in memory. In male, but not female BDNF HET mice, adolescent corticosterone treatment induced deficits in short-term spatial memory [58], consistent with an enhanced vulnerability of male BDNF HET mice to stress [59]. At a cellular level, expression of the interneuron marker, parvalbumin, was reduced in male, but not female BDNF HET mice during adolescent development [60], corresponding with reduced cell density in specific areas of the prefrontal cortex [60]. With respect to the effect of chronic METH, previous studies have shown that it enhances BDNF levels in the hippocampus of male, but not female rats [61]. Several other studies have similarly shown sex differences in BDNF signalling and effects [21,62]. Future studies on the role of BDNF in neuropsychiatric disorders will therefore have to include both males and females.

### 4.2. Chronic METH Induces Locomotor Hyperactivity Sensitisation Independent of D3 Receptors

As expected, METH pretreatment produced sensitisation of the locomotor hyperactivity induced by an acute METH challenge. This dose of METH challenge also uncovered sex-specific genotype effects in female BDNF HET and DM mice, which were more hyperactive than the WT or D3 knockout genotypes. These results are partially supported by previous studies using this escalating METH sensitisation model. Manning et al. [17] described that BDNF HET mice displayed increased amphetamine-induced hyperactivity, even in the absence of any METH pretreatment, suggesting endogenous sensitisation associated with BDNF deficiency [17]. This endogenous sensitisation is similar to that in female saline-pretreated BDNF HET and DM mice in the present study. However, unlike Manning et al. [17], we found a concomitant hypersensitivity to METH in METH-pretreated female BDNF HET mice. Although the reasons for the differences between these studies is unclear, the lack of further hypersensitivity in METH-pretreated BDNF HET mice in the study of Manning et al. [17] could reflect a locomotor hyperactivity ceiling effect in response to D-amphetamine used in that study. Hypersensitivity to psychostimulant administration in our study suggests BDNF may be implicated in mediating neuronal networks governing sensitisation development and psychosis and provides evidence for neurotrophic modulation of psychomotor behaviours.

No significant differences were observed between mice with the D3 knockout genotype (D3 knockouts and DM) and mice with the D3 receptor wildtype genotype (BDNF HET and wildtype controls). A previous study described hypersensitivity in D3 knockout mice to amphetamine at a similar dose [28]. However, it is possible that these studies are not comparable to the present results based on differences between the effects of METH and D-amphetamine, such as differential dopamine release and DAT modulation [29,63]. Therefore, our acute dose of 3 mg/kg METH may induce similar dopaminergic effects as higher amphetamine doses that were not impacted by the loss of D3 receptors in the previous study [63]. D3 receptor effects on cocaine-induced locomotor hyperactivity appear to be similarly dose-dependent in mice. A study examining locomotor hyperactivity in response to acute cocaine treatment reported a loss of significant differences between WT and D3 receptor knockout mice at higher cocaine doses [64]. This could suggest that the dampening effects of the D3 receptor activation on motor behaviours only occur at a lower dopaminergic stimulation, where small changes in extracellular DA levels could prompt a greater activity in D3 receptor-depleted systems. This effect would be masked at higher psychostimulant drug doses where the D3 receptor activation is unable to dampen dopaminergic signalling. Our data did not support any significant impact of the D3 receptor loss on locomotor hyperactivity to either low or high METH doses.

### 4.3. Sex-Specific Changes in Dopamine Receptor and Dopamine Transporter Gene Expression in BDNF HET Mice in the Striatum and Frontal Cortex

There were several changes in dopamine receptor and transporter gene expression in the striatum although these generally did not straightforwardly explain the changes in PPI regulation observed in these genotypes. Few previous studies have comprehensively assessed dopamine receptor and DAT mRNA levels following chronic METH, as was done in the present study. We found that METH pretreatment had no long-term effects on dopamine receptor expression in the striatum. In contrast, in male mice, METH pretreatment significantly reduced DAT expression in wildtype and D3 knockout mice, whereas in female mice a decrease in DAT expression was observed only in BDNF HET mice and double mutants. These findings again reveal a strictly sex-specific role of BDNF.

Independent of the effect of METH, we observed significantly reduced D1 and D2 receptor expression in male BDNF HET and DM mice compared to wildtype and D3 knockout mice. Male DM mice also had lower D2 receptor gene expression than mice with D3 receptor knockout only. Again, these changes were male-specific, as there were no differences in D1 and D2 receptor expression between the groups in female mice. As expected, D3 receptor expression was significantly reduced in D3KO and DM mice. D3 receptor gene expression was furthermore significantly lower in both male and female BDNF HET mice compared to wildtype mice, consistent with the previously described stimulatory effect of BDNF on D3 receptor expression [36,65].

Also in the frontal cortex, we found sex-specific downregulation of dopamine receptor gene expression in BDNF HET mice in the frontal cortex. For example, although there were no effects on D1 receptor gene expression in either males or females, in males, a significantly reduced D2 receptor expression was found in BDNF HET mice and DM mice compared to the combined wildtype and D3 knockout groups. In contrast there was a significant increase in female D3 receptor knockout mice and DM mice compared to wildtype and BDNF HET mice combined. While these sex-specific effects were independent of METH pretreatment, in the frontal cortex of female mice D2 receptor expression was upregulated by METH pretreatment in the D3 receptor knockout genotype but there was no METH pretreatment effect in wildtype mice and BDNF HET mice. Finally, as expected, D3 receptor gene expression in the frontal cortex was significantly reduced in D3 knockout mice and in BDNF HET mice compared to wildtype mice. Overall, these data show complex effects of genotype and METH pretreatment strongly dependent on sex.

The regulation of PPI involves a complex circuitry with both midbrain and forebrain regions interacting to (1) mediate the startle response, (2) mediate PPI, and (3) modulate PPI mediation [66]. Among the forebrain regions modulating PPI are the frontal cortex and striatum but also various other regions including the ventral hippocampus, nucleus accumbens, and amygdala [41,67]. While the present results provide insight into the long-term interactive effects of METH and BDNF on dopamine receptor and DAT expression in the striatum and frontal cortex, further studies in other brain regions are required for a more comprehensive insight into this interaction in the PPI regulatory circuitry.

### 4.4. Limitations

The mice used in the present study were tested four times for PPI, three times for locomotor (hyper)activity, and subsequently their brains were used for the analysis of dopamine receptor and DAT gene expression. It cannot be excluded that the behavioural tests, including acute drug challenges, influenced the outcome of the PCR analysis. Similarly, it is possible that testing the animals for PPI somehow influenced the outcome of the locomotor hyperactivity tests. Ideally, separate cohorts of mice should have been used for the different behavioural tests, and a separate non-behavioural cohort should have been included for analysis of the effect of METH on dopamine receptor and DAT gene expression. However, this would have required a very much larger number of animals, with the associated ethical and budgetary concerns.

We used mice with constitutive gene modifications, and it is possible that these life-long changes in BDNF and D3 expression resulted in compensatory expression changes in other neurotransmitter systems or growth factors. Future studies should therefore include BDNF and D3 receptor gene expression changes at specific later stages of the neurodevelopmental axis. Future studies should furthermore focus on other components of dopaminergic neurotransmission, such as dopamine turnover and metabolism, and differential gene expression effects in neuronal and glial cell populations.

## 5. Conclusions

These results show an interaction of BDNF with the effects of METH on the regulation of PPI. Specifically, intact BDNF signalling appears to normally protect particularly males from METH causing a dysregulation of PPI. Importantly, our findings do not support a role for D3 receptors in the effect of BDNF. Therefore, the role of these receptors in psychoses and schizophrenia remains unclear. On the other hand, several previous studies have shown reduced levels of BDNF in postmortem brain regions such as the prefrontal cortex and hippocampus [68,69,70,71], as well as deficits in PPI [41,72] in patients with schizophrenia. Treatments aimed at stimulation of the BDNF receptor, tropomyosin receptor-kinase B (TrkB), may prove clinically beneficial in counteracting the effects of METH, at least on PPI, and this may extend to psychosis and schizophrenia more generally. Indeed, recently, we showed that treatment with a TrkB receptor agonist could counteract PPI deficits in a maternal immune activation model of psychosis [73]. Moreover, in other psychosis and schizophrenia models, TrkB receptor activation has also shown promising results [74,75]. Alternatively, nonpharmacological interventions such as environmental enrichment [76] or exercise [77,78,79] may be beneficial in both schizophrenia and METH psychosis by enhancing BDNF expression in the brain. Clinical studies are now required to confirm whether enhanced neurotrophic support can be a useful treatment in psychotic disorders.

## Figures and Tables

**Table 1:** Number of mice per group.

	Chronic Saline					Chronic METH			
Genotype	WT	HET	D3KO	DM		WT	HET	D3KO	DM
Male	12	13	10	13		11	14	10	12
Female	12	15	13	14		13	11	10	13

WT = wildtype, HET = BDNF heterozygote, D3KO = D3 receptor knockout, DM = double-mutant.

## Data Availability

Data are available from the corresponding author upon request.

## Supplementary Materials

The following supporting information can be downloaded at: https://www.mdpi.com/article/10.3390/biomedicines11082290/s1, Figure S1: Breeding protocol to generate double-mutant mice; Figure S2: Timeline of the experiments; Figure S3. BDNF haploinsufficiency, but not D3 receptor knockout, alters the effect of METH pretreatment on body weight gain; Figure S4: Sex-specific minor effects of MK-801 challenge on average startle; Table S1: Mean PPI at every prepulse intensity (PP) and average (Avg) of all PPs during saline, apomorphine (APO) and MK-801 challenge sessions. References cited in supplementary file [47,50,80,81,82,83,84,85].
