# Supplementary material for: Interaction of Brain-Derived Neurotrophic Factor with the Effects of Chronic Methamphetamine on Prepulse Inhibition in Mice Is Independent of Dopamine D3 Receptors
Source: Biomedicines. 2023 Aug 17;11(8):2290. doi: 10.3390/biomedicines11082290 (PMC10452514; doi:10.3390/biomedicines11082290)
Supplement: Supplementary file 1 [file biomedicines-11-02290-s001.zip › biomedicines-2560052-supplementary.pdf]

Supplementary Materials

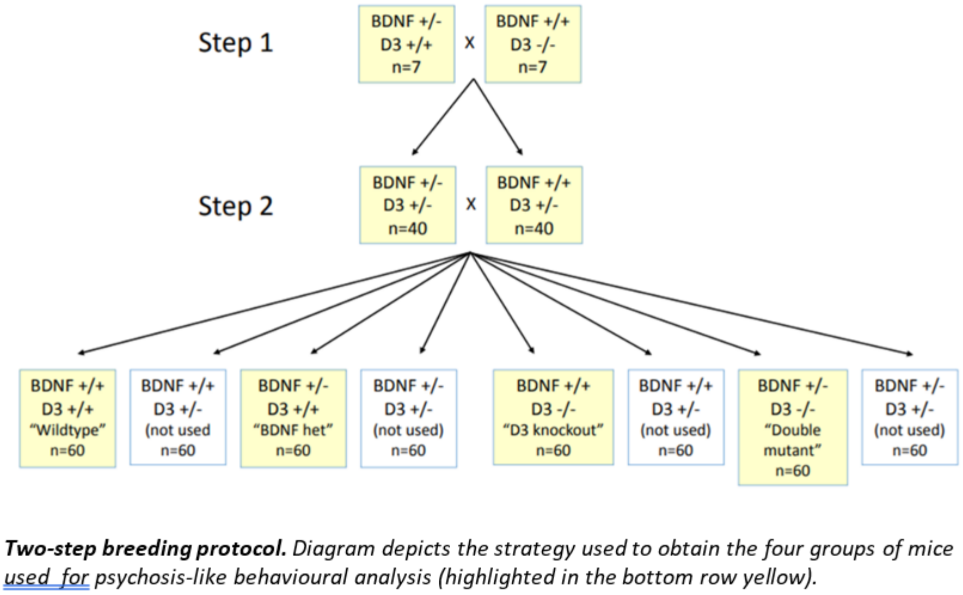

Figure S1. Breeding protocol to generate double-mutant mice.

Note: We did not conduct a SHIRPA (Rogers et al. 1997 [1]) screen or physical exam of the double-mutants because the BDNF HET/ D3KO double-mutant model was generated by a cross of two previously established mouse lines which have been examined extensively for basic motor functions and behavioural abnormalities. BDNF HET do not exhibit overt behavioural changes or other developmental or reproductive deficits or predisposition to disease. One abnormality reported in this study and others is a small but significant increase in average animal body weight in BDNF HET mice. In addition, this strain requires no additional care and maintains regular breeding activity when crossed with WT mice (Ernfors et al. 1994 [2]). Previous behavioural analysis of the D3KO mouse strain has demonstrated reduced anxiety levels in open field, forced swim test, and elevated plus maze paradigms (Moraga-Amaro et al. 2014 [3], Steiner et al. 1997 [4]) but mixed results regarding increased basal locomotor activity (Accili et al. 1996 [5], Moraga-Amaro et al. 2014 [3]). However, another study reported no differences between D3KO and WT mice in a standardised behaviour battery, including exploratory behaviour and anxiety and depressive-like symptoms (Chourbaji et al. 2008 [6]).

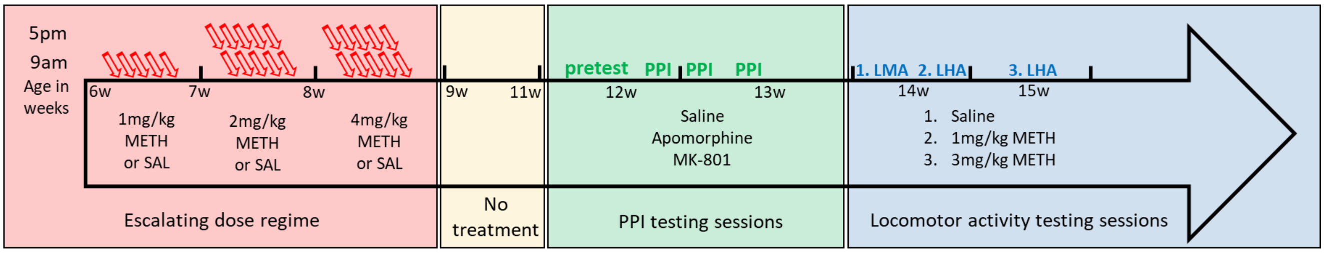

Figure S2. Timeline of the experiments.

Mice were treated daily with different doses of METH (see Methods section for details) and, after a no-treatment period of two weeks, were tested for changes in the regulation of PPI and locomotor activity (LMA) and drug-induced locomotor hyperactivity (LHA).

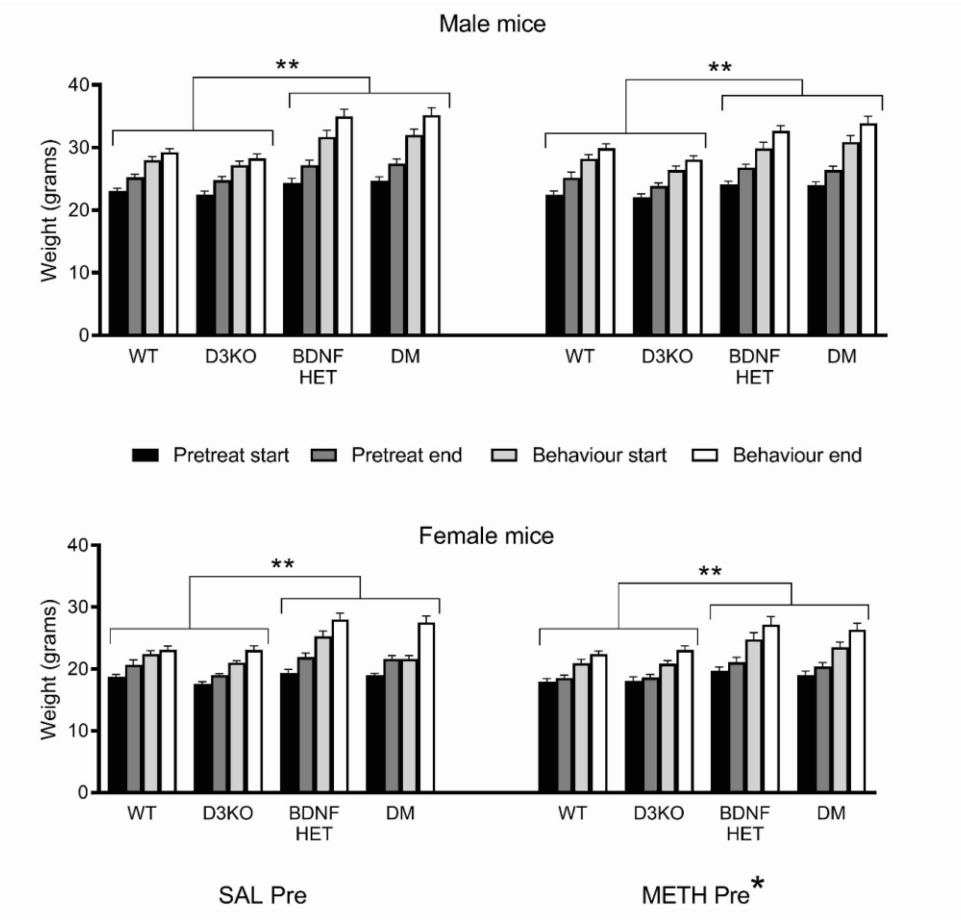

Body weight of mice across the study. *Mice were pretreated with either saline (SAL Pre) or METH (METH Pre). Males were significantly heavier than females (not indicated) and BDNF HET mice and DM mice were significantly heavier than WT and D3KO mice (** p < 0.05). METH pretreatment slightly altered body weight gain depending on BDNF genotype (* p < 0.05). For other comparisons, see text.*

Figure S3. BDNF haploinsufficiency, but not D3 receptor knockout, alters the effect of METH pretreatment on body weight gain.

Mice were weighed twice per week during METH or saline pretreatment and once before every acute drug IP injection. Bodyweight was analysed at 4 timepoints: start of the pretreatment phase (Pretreat start), end of pretreatment phase (Pretreat end), end of no-treatment phase (Behaviour start), and end of behavioural analyses (Behaviour end).

As expected, all mice increased their body weight over the course of the treatment and behavioural testing period (main effect of time, $F(2.002, 354.3) = 1308.5$, $p < 0.001$, $\eta p^2 = 0.881$) and male mice were heavier and gained more weight than female mice (main effect of sex, $F(1,177) = 284.9$, $p < 0.001$, $\eta p^2 = 0.617$; sex × time interaction, $F(2.002, 354.3) = 21.94$, $p < 0.001$, $\eta p^2 = 0.110$). There was also a genotype effect (main effect, $F(3,177) = 26.21$, $p < 0.001$, $\eta p^2 = 0.308$; genotype × time interaction $F(6.005, 354.3) = 25.45$; $p < 0.001$, $\eta p^2 = 0.301$) with further analysis by BDNF genotype confirming that BDNF HET and double-mutant mice had higher bodyweights and greater body weight gain than wildtype and D3 knockout mice (main effect of BDNF genotype, $F(1,177) = 77.27$, $p < 0.001$, $\eta p^2 = 0.304$; BDNF genotype × time interaction, $F(2.002, 354.3) = 75.30$, $p < 0.001$, $\eta p^2 = 0.298$).

METH pretreatment affected body weight gain dependent on BDNF genotype (pretreatment × genotype × time interaction, $F(2.002, 354.3) = 4.933$, $p = 0.008$, $\eta p^2 = 0.027$). Although all groups gained weight over the course of the experiment, METH pretreatment slightly attenuated this in the combined BDNF HET and DM group (difference from last day of behavioural testing minus first day of pretreatment 10.53 ± 0.59g in saline-

pretreated males vs. 9.13 ± 0.53g in METH-pretreated males, 8.54 ± 0.49g in saline-pretreated females vs. 7.36 ± 0.57g in METH-pretreated females, F(2.42, 195.6) = 5.365, $p < 0.001$). In contrast, body weight gain was slightly greater following METH pretreatment in the combined wildtype and D3 knockout mice groups (difference from last day of behavioural testing minus first day of pretreatment 4.44 ± 0.18g in saline-pretreated females vs. 4.67 ± 0.24g in METH-pretreated females, 6.02 ± 0.24g in saline-pretreated males vs. 6.77 ± 0.35g in METH-pretreated males, F(1.685, 161.7) = 3.86, $p = 0.030$, Figure S3).

The observation, that BDNF HET mice gained significantly more weight over the duration of the study, is in line with previous research demonstrating the role of BDNF in regulating eating behaviours in mice (Kernie et al. 2000 [7]). D3 receptor loss did not affect body weight at any time point and saline- and METH-pretreated mice gained weight at equal rates. These data that the escalating METH-pretreatment regimen is not having substantial or persistent neurotoxic/anorexic effects. Other mouse model studies with comparable dosages have reported similar findings (Davidson et al. 2005 [8]).

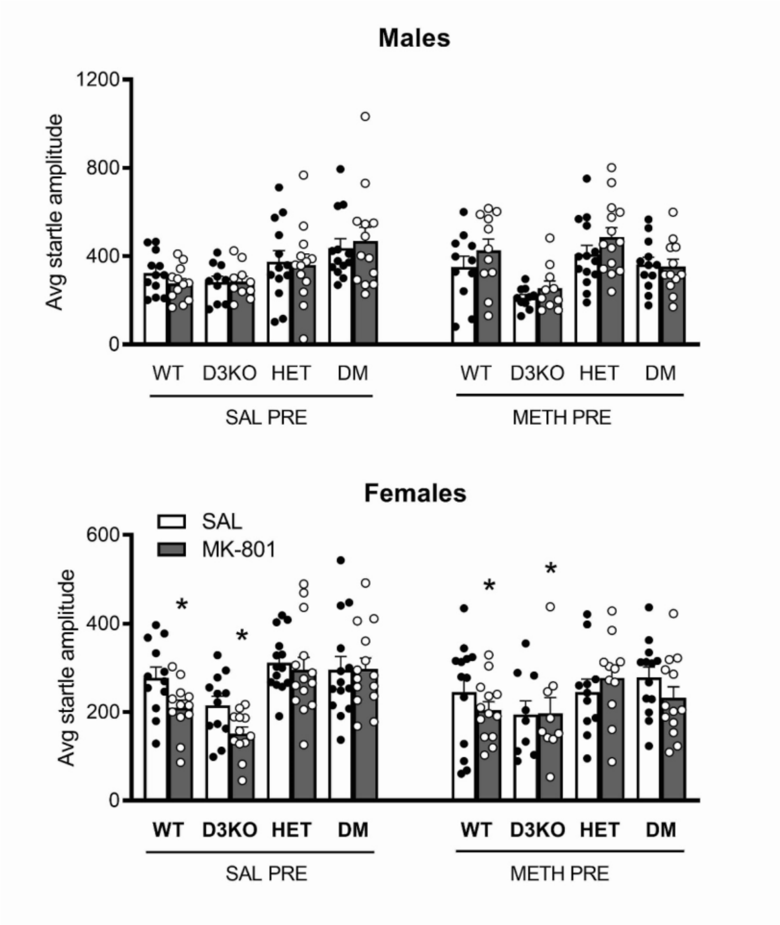

*__Average startle__ in male (top panel) and female (bottom panel) wildtype (WT), D3 receptor knockout (D3KO), BDNF heterozygous mice (HET) and double-mutant mice with both BDNF heterozygosity and D3 knockout (DM). The mice were pretreated with a three-week injection regimen of methamphetamine (METH Pre) or saline (Sal Pre). Two weeks later, startle was measured at baseline following saline injection (SAL) or MK-801. * p < 0.05 for difference between startle following MK-801 challenge and following saline in WT and D3KO but not BDNF HET and DM mice. For details of analysis see main text.*

Figure S4. Sex-specific minor effects of MK-801 challenge on average startle.

Table S1. Mean PPI at every prepulse intensity (PP) and average (Avg, in **bold**) of all PPs during saline, apomorphine (APO) and MK-801 challenge sessions. PPI was measured at 30 and 100ms inter-stimulus interval (ISI).

A: Male Saline Pretreatment

	30ms ISI					100ms ISI				
	**PP2**	**PP4**	**PP8**	**PP16**	**Avg**	**PP2**	**PP4**	**PP8**	**PP16**	**Avg**
**Male wildtype saline pretreatment**										
SAL	7.3 ± 7.6	18.4 ±4.4	41.8 ± 5.5	59.4 ± 4.1	**31.7 ± 3.1**	6.8 ± 13.5	21.9 ± 5.9	41.2 ± 3.2	58.4 ± 4.2	**32.1 ± 3.8**
APO	−13.4 ± 10.0	−17.4 ± 6.7	5.2 ± 8.3	28.1 ± 11.4	**0.6 ± 5.7**	−17.2 ± 9.2	3.9 ± 7.1	19.7 ± 8.5	42.9 ± 7.6	**12.3 ± 4.1**
MK-801	−2.1 ± 4.8	−7.3 ± 5.6	11.0 ± 6.0	40.2 ± 4.5	**10.4 ± 2.8**	2.7 ± 4.7	−6.1 ± 3.8	11.7 ± 5.9	28.4 ± 5.4	**9.2 ± 2.6**
**Male D3 knockout saline pretreatment**										
SAL	−0.6 ± 12.7	27.4 ± 10.7	37.6 ± 13.7	66.1 ± 6.7	**32.6 ± 8.9**	9.5 ± 8.1	15.6 ± 8.1	34.4 ± 10.0	52.4 ± 7.8	**28.0 ± 6.8**
APO	−17.9 ± 9.0	−17.0 ± 13.8	8.4 ± 18.0	52.8 ± 8.1	**6.6 ± 8.9**	0.8 ± 6.0	−3.3 ± 8.1	38.1 ± 7.1	58.5 ± 4.6	**23.5 ± 4.1**
MK-801	-4.9 ± 4.8	7.9 ± 10.0	24.5 ± 5.8	46.1 ± 7.9	**18.4 ± 4.7**	-0.7 ± 10.7	1.0 ± 5.5	1.1 ± 13.5	20.3 ± 11.9	**5.4 ± 7.7**
**Male BDNF HET saline pretreatment**										
SAL	2.6 ± 6.9	11.9 ± 5.0	29.9 ± 5.5	49.4 ± 5.1	**23.4 ± 4.3**	-2.8 ± 5.4	12.2 ± 4.5	31.8 ± 4.3	53.3 ± 4.6	**23.6 ± 2.5**
APO	-8.3 ± 8.4	-5.9 ± 5.3	-4.2 ± 5.5	20.2 ± 5.4	**0.4 ± 3.9**	-8.3 ± 8.8	-4.3 ± 6.3	17.7 ± 3.8	45.9 ± 5.1	**12.8 ± 4.9**
MK-801	-1.7 ± 3.6	-12.0 ± 6.1	-1.1 ± 9.2	32.8 ± 5.6	**4.5 ± 3.9**	-4.7 ± 4.6	-1.2 ± 4.2	1.1 ± 3.2	24.9 ± 5.5	**5.0 ± 3.2**
**Male DM saline pretreatment**										
SAL	0.1 ± 6.8	23.3 ± 5.7	42.0 ± 4.4	54.8 ± 2.7	**30.1 ± 3.7**	15.3 ± 3.4	24.7 ± 3.9	39.5 ± 2.8	53.8 ± 4.1	**33.3 ± 2.4**
APO	-9.5 ± 6.8	-6.4 ± 6.9	18.1 ± 5.2	33.9 ± 7.6	**9.0 ± 5.4**	3.0 ± 3.8	0.8 ± 4.8	22.1 ± 8.8	58.1 ± 6.0	**21.0 ± 4.1**
MK-801	-5.7 ± 5.4	-10.5 ± 8.2	9.0 ± 7.3	45.2 ± 5.6	**9.5 ± 5.4**	2.8 ± 5.1	3.2 ± 7.8	16.8 ± 7.6	27.9 ± 6.1	**12.7 ± 5.3**

B: Male Meth Pretreatment

	30ms ISI					100ms ISI				
	**PP2**	**PP4**	**PP8**	**PP16**	**Avg.**	**PP2**	**PP4**	**PP8**	**PP16**	**Avg.**
**Male wildtype METH pretreatment**										
SAL	11.2 ± 7.6	17.8 ± 4.0	38.5 ± 4.9	55.4 ± 6.5	**30.7 ± 3.3**	6.4 ± 5.6	24.8 ± 7.3	42.2 ± 4.1	54.8 ± 2.6	**32.0 ± 3.0**
APO	−11.9 ± 6.6	−9.1 ± 14.4	0.8 ± 7.5	31.9 ± 9.9	**3.0 ± 7.8**	−22.3 ± 11.6	4.3 ± 7.8	23.9 ± 6.0	48.4 ± 6.4	**13.6 ± 6.2**
MK-801	10.2 ± 9.6	16.9 ± 5.7	21.4 ± 7.5	39.4 ± 5.6	**22.0 ± 5.3**	3.3 ± 9.4	4.0 ± 6.6	8.4 ± 7.9	31.1 ± 6.7	**11.7 ± 5.9**
**Male D3 knockout METH pretreatment**										
SAL	−12.3 ± 9.5	19.6 ± 13.6	44.2 ± 5.5	77.5 ± 2.8	**32.3 ± 6.2**	18.7 ± 6.5	38.2 ± 5.4	38.6 ± 7.0	71.4 ± 3.0	**41.7 ± 3.7**
APO	−32.7 ± 7.9	3.9 ± 10.8	18.7 ± 15.0	52.8 ± 11.6	**10.7 ± 9.8**	−17.2 ± 7.9	−3.3 ± 9.8	28.9 ± 7.9	55.1 ± 7.5	**15.96.5**
MK-801	−7.7 ± 12.3	−7.0 ± 8.9	16.6 ± 7.3	45.4 ± 6.2	**11.8 ± 6.5**	−3.8 ± 5.3	3.0 ± 4.7	8.0 ± 7.8	38.4 ± 6.4	**11.4 ± 3.4**
**Male BDNF HET METH pretreatment**										
SAL	3.9 ± 7.8	16.4 ± 6.3	20.1 ± 6.1	47.6 ± 4.8	**22.0 ± 4.7**	4.0 ± 5.8	20.7 ± 4.4	34.3 ± 2.9	50.8 ± 4.1	**27.4 ± 3.1**
APO	−24.2 ± 7.8	−9.8 ± 6.4	6.6 ± 7.6	30.6 ± 7.2	**0.8 ± 5.4**	1.1 ± 5.0	−2.7 ± 5.2	18.7 ± 5.3	50.7 ± 3.7	**16.9 ± 3.1**
MK-801	7.2 ± 5.6	15.0 ± 4.3	26.3 ± 3.1	49.2 ± 3.3	**24.4 ± 2.2**	5.4 ± 4.1	9.3 ± 5.3	23.5 ± 4.4	34.7 ± 2.4	**18.2 ± 2.6**
**Male DM METH pretreatment**										
SAL	−4.3 ± 8.1	19.7 ± 4.7	30.0 ± 4.3	51.5 ± 6.2	**24.2 ± 4.5**	11.1 ± 6.5	9.2 ± 6.2	28.4 ± 5.2	46.9 ± 4.6	**23.9 ± 4.3**
APO	−3.7 ± 6.2	−5.5 ± 10.4	14.5 ± 9.7	40.0 ± 7.9	**11.3 ± 7.9**	−2.0 ± 5.1	5.0 ± 5.8	34.5 ± 5.5	51.5 ± 5.1	**22.3 ± 3.6**
MK-801	0.0 ± 5.7	3.1 ± 9.0	22.3 ± 7.7	42.9 ± 5.8	**17.1 ± 5.3**	12.2 ± 4.5	13.4 ± 4.4	10.4 ± 9.5	36.9 ± 4.3	**18.2 ± 4.6**

C: Female Saline Pretreatment

	30ms ISI					100ms ISI				
	**PP2**	**PP4**	**PP8**	**PP16**	**Avg.**	**PP2**	**PP4**	**PP8**	**PP16**	**Avg.**
**Female wildtype saline pretreatment**										
SAL	10.0 ± 3.5	26.5 ± 5.7	42.0 ± 5.3	64.8 ± 4.0	**35.8 ± 2.8**	15.3 ± 4.6	25.3 ± 4.7	33.1 ± 4.6	56.0 ± 3.7	**32.4 ± 3.3**
APO	−16.1 ± 8.3	−5.6 ± 11.6	3.4 ± 6.6	34.8 ± 6.4	**4.1 ± 5.3**	−4.6 ± 7.9	−1.0 ± 7.2	31.1 ± 4.7	50.9 ± 5.0	**19.1 ± 4.3**
MK-801	−9.9 ± 8.7	−6.3 ± 6.2	14.7 ± 5.8	37.4 ± 3.8	**9.0 ± 3.4**	−9.7 ± 5.9	−1.6 ± 5.9	8.8 ± 3.1	24.2 ± 7.1	**5.4 ± 2.5**
**Female D3 knockout saline pretreatment**										
SAL	5.6 ± 5.2	18.5 ± 6.7	35.4 ± 3.9	67.5 ± 3.1	**31.8 ± 3.8**	3.8 ± 5.0	27.0 ± 7.0	43.7 ± 5.6	61.9 ± 3.2	**34.1 ± 3.7**
APO	−22.2 ± 12.5	6.6 ± 7.0	13.5 ± 6.2	40.0 ± 8.1	**9.5 ± 6.9**	−13.5 ± 7.1	4.5 ± 7.1	15.0 ± 8.8	50.4 ± 4.9	**14.1 ± 4.4**
MK-801	−20.6 ± 20.2	−8.3 ± 14.9	6.8 ± 13.0	24.4 ± 8.6	**0.6 ± 13.3**	−6.5 ± 10.6	−3.2 ± 8.3	2.7 ± 11.3	19.3 ± 7.4	**3.1 ± 6.9**
**Female BDNF HET saline pretreatment**										
SAL	5.6 ± 4.8	30.4 ± 5.0	33.5 ± 3.3	54.9 ± 4.3	**31.1 ± 2.2**	12.3 ± 4.6	15.1 ± 3.6	37.5 ± 3.6	55.3 ± 3.9	**30.1 ± 2.6**
APO	−17.8 ± 5.9	−5.5 ± 8.5	6.8 ± 4.8	9.9 ± 13.3	**−1.7 ± 5.7**	6.4 ± 4.3	−6.1 ± 5.5	14.5 ± 4.4	46.4 ± 4.2	**15.3 ± 3.0**
MK-801	−5.3 ± 6.8	6.0 ± 6.2	14.5 ± 7.1	39.2 ± 4.3	**13.6 ± 4.8**	−13.6 ± 9.6	−13.6 ± 9.1	10.0 ± 6.4	28.8 ± 4.3	**2.9 ± 5.8**
**Female DM saline pretreatment**										
SAL	−1.3 ± 6.1	8.8 ± 5.9	35.5 ± 4.4	46.9 ± 3.0	**22.5 ± 3.6**	3.4 ± 3.5	13.4 ± 3.0	33.2 ± 2.3	48.6 ± 3.6	**24.6 ± 1.7**
APO	−21.1 ± 8.7	−17.0 ± 9.0	7.3 ± 10.7	29.8 ± 5.7	**−0.2 ± 6.6**	−1.4 ± 6.6	2.3 ± 5.5	16.4 ± 6.6	48.0 ± 3.6	**16.3 ± 3.8**
MK-801	−1.9 ± 5.6	−2.0 ± 5.6	18.2 ± 5.8	38.6 ± 5.1	**13.2 ± 3.9**	2.1 ± 4.0	−5.1 ± 6.0	7.1 ± 5.7	28.2 ± 4.4	**8.1 ± 3.9**

D: Female METH Pretreatment										
	30ms ISI					100ms ISI				
	PP2	PP4	PP8	PP16	Avg.	PP2	PP4	PP8	PP16	Avg.
**Female wildtype METH pretreatment**										
SAL	−3.3 ± 8.8	24.0 ± 4.6	36.4 ± 5.9	61.0 ± 3.1	**29.5 ± 3.1**	10.6 ± 6.5	20.2 ± 3.5	34.7 ± 3.5	54.3 ± 3.0	**29.9 ± 2.7**
APO	−20.0 ± 10.8	−2.1 ± 10.1	11.9 ± 7.1	35.8 ± 5.6	**6.4 ± 5.3**	−7.6 ± 8.9	11.0 ± 4.6	18.8 ± 5.6	49.0 ± 5.3	**17.8 ± 3.6**
MK-801	0.3 ± 7.5	2.2 ± 5.6	19.9 ± 4.8	34.8 ± 5.6	**14.3 ± 4.0**	0.0 ± 5.8	7.2 ± 6.7	7.0 ± 5.9	23.7 ± 7.2	**9.5 ± 4.5**
**Female D3 knockout METH pretreatment**										
SAL	15.2 ± 5.7	26.0 ± 7.2	40.5 ± 6.1	72.1 ± 2.4	**38.4 ± 3.5**	15.3 ± 5.9	19.6 ± 4.2	49.1 ± 6.7	61.6 ± 5.6	**36.4 ± 3.4**
APO	−24.8 ± 19.9	−9.4 ± 8.4	27.6 ± 8.4	49.1 ± 9.0	**10.6 ± 6.8**	−1.2 ± 11.9	11.3 ± 11.3	28.5 ± 4.4	55.7 ± 5.0	**23.6 ± 5.2**
MK-801	−4.3 ± 18.0	14.4 ± 11.5	23.2 ± 9.0	39.1 ± 11.6	**18.1 ± 11.1**	14.5 ± 9.5	4.6 ± 6.6	18.8 ± 10.0	33.5 ± 10.9	**17.8 ± 7.3**
**Female BDNF HET METH pretreatment**										
SAL	9.0 ± 8.3	17.9 ± 8.0	31.4 ± 7.5	57.1 ± 6.4	**28.9 ± 6.4**	6.6 ± 5.3	25.8 ± 5.6	38.9 ± 5.5	60.1 ± 4.1	**32.8 ± 4.0**
APO	−18.4 ± 15.3	−14.7 ± 11.6	8.5 ± 10.6	22.9 ± 13.3	**−0.4 ± 10.8**	−3.6 ± 9.8	−5.3 ± 8.7	17.3 ± 8.6	41.4 ± 8.3	**12.4 ± 7.0**
MK-801	−6.5 ± 14.0	−1.4 ± 8.8	14.0 ± 9.6	40.4 ± 7.3	**11.6 ± 7.7**	0.0 ± 6.7	6.8 ± 10.3	22.4 ± 6.3	37.2 ± 7.5	**16.6 ± 5.5**
**Female DM METH pretreatment**										
SAL	6.7 ± 4.5	32.1 ± 5.0	33.5 ± 4.6	56.0 ± 4.2	**32.1 ± 2.9**	11.8 ± 4.6	25.1 ± 5.1	36.3 ± 3.8	59.8 ± 2.4	**33.2 ± 2.2**
APO	−12.2 ± 7.0	−8.6 ± 7.5	13.4 ± 6.6	22.7 ± 8.6	**3.8 ± 5.8**	−4.1 ± 4.8	−1.6 ± 7.2	19.3 ± 6.8	42.2 ± 3.0	**14.0 ± 3.2**
MK-801	−1.1 ± 6.2	−7.3 ± 8.8	14.9 ± 7.2	38.8 ± 7.5	**11.3 ± 6.3**	5.2 ± 7.4	−2.5 ± 6.9	15.1 ± 4.3	24.9 ± 6.6	**10.7 ± 3.6**

References

1. Rogers, D.C.; Fisher, E.M.; Brown, S.D.; Peters, J.; Hunter, A.J.; Martin, J.E. Behavioral and functional analysis of mouse phenotype: SHIRPA, a proposed protocol for comprehensive phenotype assessment. *Mamm. Genome* **1997**, *8*, 711–713. https://doi.org/10.1007/s003359900551.
2. Ernfors, P.; Lee, K.F.; Jaenisch, R. Mice lacking brain-derived neurotrophic factor develop with sensory deficits. *Nature* **1994**, *368*, 147–150. https://doi.org/10.1038/368147a0.
3. Moraga-Amaro, R.; Gonzalez, H.; Pacheco, R.; Stehberg, J. Dopamine receptor D3 deficiency results in chronic depression and anxiety. *Behav. Brain Res.* **2014**, *274*, 186–193. https://doi.org/10.1016/j.bbr.2014.07.055.
4. Steiner, H.; Fuchs, S.; Accili, D. D3 dopamine receptor-deficient mouse: evidence for reduced anxiety. *Physiol. Behav.* **1997**, *63*, 137–141. https://doi.org/10.1016/s0031-9384(97)00430-7.
5. Accili, D.; Fishburn, C.S.; Drago, J.; Steiner, H.; Lachowicz, J.E.; Park, B.H.; Gauda, E.B.; Lee, E.J.; Cool, M.H.; Sibley, D.R.; et al. A targeted mutation of the D3 dopamine receptor gene is associated with hyperactivity in mice. *Proc. Natl. Acad. Sci. USA* **1996**, *93*, 1945–1949. https://doi.org/10.1073/pnas.93.5.1945.
6. Chourbaji, S.; Brandwein, C.; Vogt, M.A.; Dormann, C.; Mueller, R.; Drescher, K.U.; Gross, G.; Gass, P. Dopamine receptor 3 (D3) knockout mice show regular emotional behaviour. *Pharmacol. Res.* **2008**, *58*, 302–307. https://doi.org/10.1016/j.phrs.2008.09.002.
7. Kernie, S.G.; Liebl, D.J.; Parada, L.F. BDNF regulates eating behavior and locomotor activity in mice. *Embo J.* **2000**, *19*, 1290–1300. https://doi.org/10.1093/emboj/19.6.1290.
8. Davidson, C.; Lee, T.H.; Ellinwood, E.H. Acute and chronic continuous methamphetamine have different long-term behavioral and neurochemical consequences. *Neurochem. Int.* **2005**, *46*, 189–203. https://doi.org/10.1016/j.neuint.2004.11.004.
